# Supplementary material for: A ZIP1 separation-of-function allele reveals that centromere pairing drives meiotic segregation of achiasmate chromosomes in budding yeast
Source: PLoS Genet. 2018 Aug 9;14(8):e1007513. doi: 10.1371/journal.pgen.1007513 (PMC6103513; doi:10.1371/journal.pgen.1007513)
Supplement: S1 Fig — (PDF) [file pgen.1007513.s001.pdf]

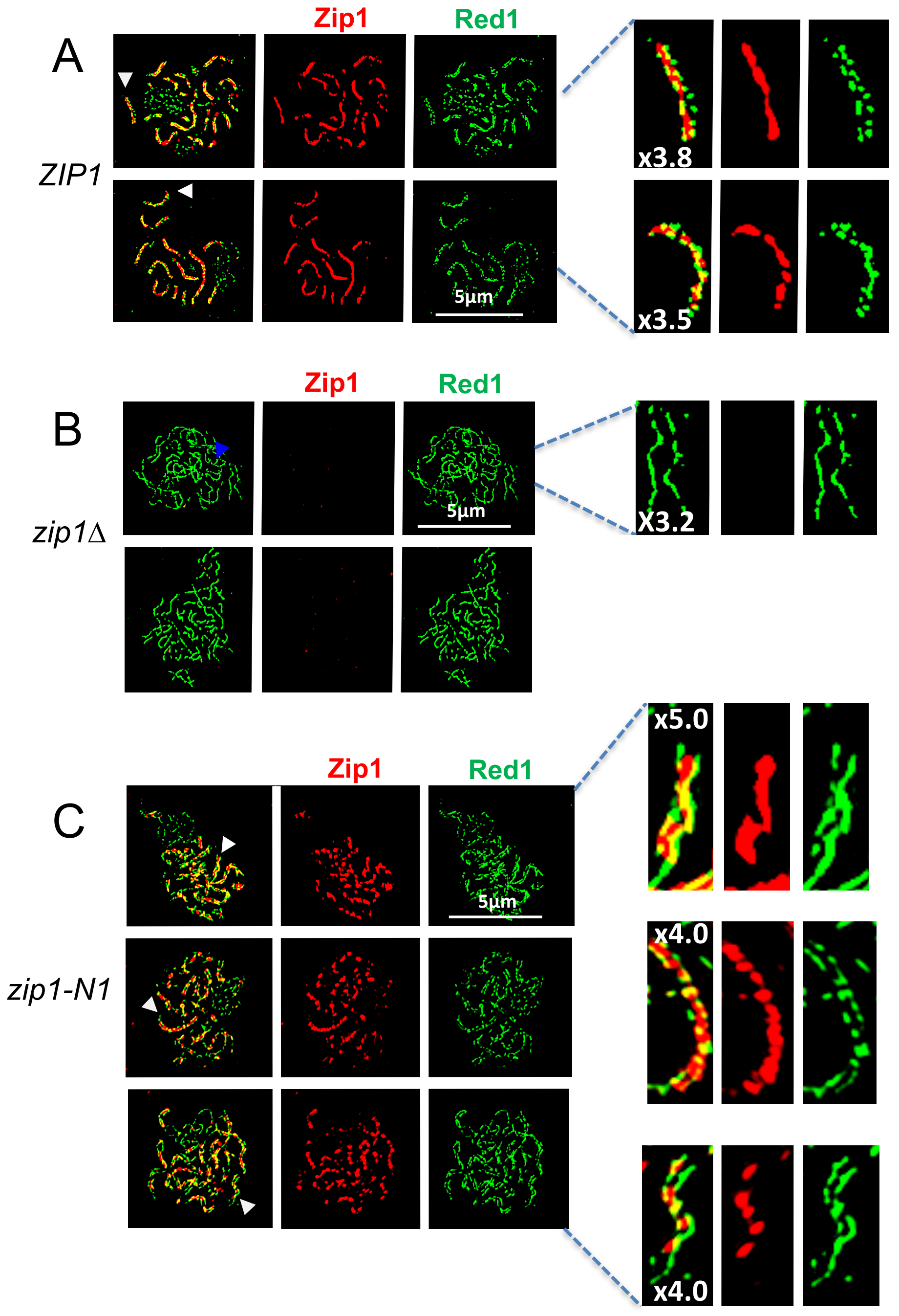

Kurdzo et al., Fig. S1

Figure S1. *zip1-N1* cells assemble synaptonemal complexes and exhibit high spore viability. Chromosome spreads were prepared from cells 5 hours after placing the cultures in sporulation medium and stained as described in Materials and Methods. The axial element protein is shown in green and Zip1 is shown in Red. Each panel presents representative spreads from **A.** *ZIP1*, **B.** *zip1Δ,* and **C.** *zip1-N1* strains. Panels to the right are larger images of individual chromosomes. The results obtained using our strains are in keeping with the more comprehensive previous study of SC assembly in *zip1-N1* mutants (Tung and Roeder, 1998) in that the *zip1-N1* strain exhibited slightly less continuous Zip1 staining in pachytene-like spreads than was observed with the wild-type control strain. It is not clear if this reflects a slight reduction in assembly kinetics, or reduced continuity of the Zip1 in the mature SC of the *zip1-N1* strain.
