# Supplementary material for: A ZIP1 separation-of-function allele reveals that centromere pairing drives meiotic segregation of achiasmate chromosomes in budding yeast
Source: PLoS Genet. 2018 Aug 9;14(8):e1007513. doi: 10.1371/journal.pgen.1007513 (PMC6103513; doi:10.1371/journal.pgen.1007513)
Supplement: S2 Fig — (PDF) [file pgen.1007513.s002.pdf]

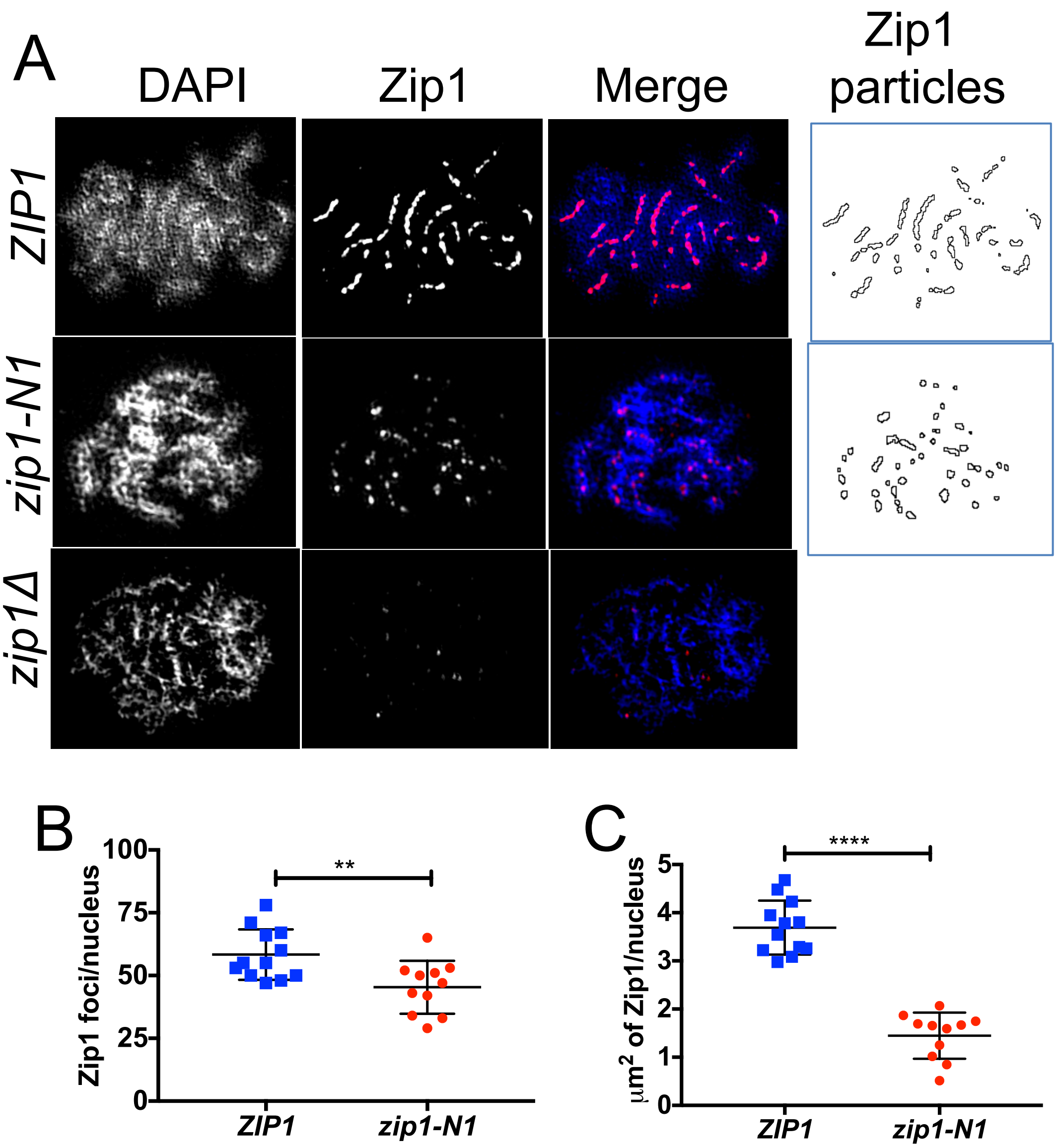

D

Spore viability

ZIP1: 40 of 48 spores (83%)
zip1-N1: 74 of 96 spores (77%)

Kurdzo et al., Fig. S2

Figure S2. Quantifying Zip1 deposition in synaptonemal complexes of *zip1-N1* mutants. Cells from wild-type, *zip1Δ,* and *zip1-N1* strains were harvested 5 hrs after meiotic induction. **A.** Chromosome spreads were prepared and stained with primary antibodies against Zip1 (Rabbit anti-Zip1, 1:1000) and Alexa 568 Goat anti-Rabbit (1:1000) secondary antibodies. Images were acquired using a DeltaVision OMX structured illumination system. Pachytene spreads (based on condensed chromosome morphology detected by DAPI staining were chosen for Zip1 quantification (A, DAPI). ImageJ software was used to analysis Zip1 amounts and distributions on the spreads, similar to approaches described previously by Humphryes et al., 2013. To analyze each spread, the Zip1 (Alexa 568) channel was converted into binary (see examples in panel **A**, Zip1 particles) then used the "Analyze Particles" function in imageJ to measure the total number of Zip1 foci in each spread (**B**), and the total area of Zip1 staining in each spread (**C**). Statistical analysis was performed using the unpaired t test in Prizm 7.0. *ZIP1* (n=11), *zip1-N1* (n=12). **p<0.01, ****p<0.0001. **D.** Tetrads were dissected to assess spore viability in *ZIP1* and *zip1-N1* strains. Though in this sample set the *zip1-N1* exhibited slightly lower spore viability than the wild-type control, as in prior studies (Tung and Roeder, 1998) there was no significant difference (Fisher's exact test, P=0.83).
