# Supplementary material for: A ZIP1 separation-of-function allele reveals that centromere pairing drives meiotic segregation of achiasmate chromosomes in budding yeast
Source: PLoS Genet. 2018 Aug 9;14(8):e1007513. doi: 10.1371/journal.pgen.1007513 (PMC6103513; doi:10.1371/journal.pgen.1007513)
Supplement: S3 Fig — (PDF) [file pgen.1007513.s003.pdf]

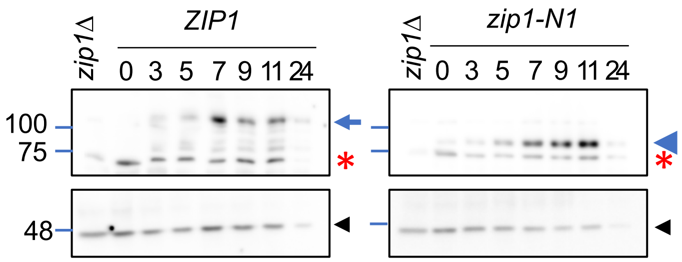

Kurdzo et al., Fig. S3

Figure S3. Western blot analysis of Zip1 and Zip1-N1 expression. Cultures were propagated and protein samples prepared as described in the Materials and Methods section. Each gel contains a sample from a *zip1Δ* meiotic culture (7 hr timepoint) and a series of samples from a meiotic time course harvested at the times indicated (in hours after meiotic induction). Western blots were probed with a rabbit antibody raised against a C-terminal region of Zip1 that is common to both wild-type and Zip1 and Zip1-N1 (top panels). Hash marks on the left indicate the mobility of size markers run on these gels (not shown). The blue arrow indicates the mobility of wild-type Zip1 protein (100kD). The blue arrowhead indicates the mobility of Zip1-N1 (84kD). The red asterisk marks the position of a non-specific band. Duplicate blots were probed with anti-Pgk1 antibodies (bottom panels). The black arrowhead indicates the position of Pgk1.
