# Supplementary material for: A ZIP1 separation-of-function allele reveals that centromere pairing drives meiotic segregation of achiasmate chromosomes in budding yeast
Source: PLoS Genet. 2018 Aug 9;14(8):e1007513. doi: 10.1371/journal.pgen.1007513 (PMC6103513; doi:10.1371/journal.pgen.1007513)
Supplement: S1 Table — (PDF) [file pgen.1007513.s004.pdf]

Table S1

Diploid	Haploids Used	Figure
DEK137	X2066: MAT**a**, trp1-63, his3-Δ1, leu2, met13-d, ura3-52::pAFS152[URA3 P_{CYC1}-GFP-lacI], MTW1-13xMYC-TRP1, tyr1-1, lys2::pMDE798[P_{DMC1}-GFP-lacI, LYS2], zip1::KanMX6, spo11::KanMX6, CEN1::pJN2[lacO256 LEU2]	**Fig. 2A**
	Y1882: MAT∝, leu2, lys2::pMDE798[P_{DMC1}-GFP-lacI], met13-c, tyr1-2, trp1-Δ63, cyh2-1, spo11::KanMX6, ura3-1, MTW1-13xMYC-his5, his3-Δ1, zip1::pELK10[ZIP1], CEN1::pJN2[lacO256 LEU2]	
DEK196	X2065: MAT**a**, trp1-63, his3-Δ1, leu2, met13-d, ura3-52::pAFS152[URA3 P_{CYC1}-GFP-lacI], MTW1-13xMYC-TRP1, tyr1-1, lys2::pMDE798[P_{DMC1}-GFP-lacI, LYS2], zip1::KanMX6, spo11::KanMX6, CEN1::pJN2[lacO256 LEU2]	**Fig. 2B**
	Y1882: MAT∝, leu2, lys2::pMDE798[P_{DMC1}-GFP-lacI], met13-c, tyr1-2, trp1-Δ63, cyh2-1, spo11::KanMX6, ura3-1, MTW1-13xMYC-his5, his3-Δ1, zip1::pELK10[ZIP1], CEN1::pJN2[lacO256 LEU2]	
DEK182	X2066: MAT**a**, trp1-63, his3-Δ1, leu2, met13-d, ura3-52::pAFS152[URA3 P_{CYC1}-GFP-lacI], MTW1-13xMYC-TRP1, tyr1-1, lys2::pMDE798[P_{DMC1}-GFP-lacI, LYS2], zip1::KanMX6, spo11::KanMX6, CEN1::pJN2[lacO256 LEU2]	**Fig. 2B**
	Y1931: MAT∝, leu2, lys2::pMDE798[P_{DMC1}-GFP-lacI], met13-c, tyr1-2, trp1-Δ63, cyh2-1, spo11::KanMX6, ura3-1, MTW1-13xMYC-his5, his3-Δ1, zip1::pELK11[zip1-N1], CEN1::pJN2[lacO256 LEU2]	
DEK208	X2065: MAT**a**, trp1-63, his3-Δ1, leu2, met13-d, ura3-52::pAFS152[URA3 P_{CYC1}-GFP-lacI], MTW1-13xMYC-TRP1, tyr1-1, lys2::pMDE798[P_{DMC1}-GFP-lacI, LYS2], zip1::KanMX6, spo11::KanMX6, CEN1::pJN2[lacO256 LEU2]	**Fig. 2B**
	Y1931: MAT∝,leu2, lys2::pMDE798[P_{DMC1}-GFP-lacI], met13-c, tyr1-2, trp1-Δ63, cyh2-1, spo11::KanMX6, ura3-1, MTW1-13xMYC-his5, his3-Δ1, zip1::pELK11[zip1-N1], CEN1::pJN2[lacO256 LEU2]	
DEK197	X2065: MAT**a**, trp1-63, his3-Δ1, leu2, met13-d, ura3-52::pAFS152[URA3 P_{CYC1}-GFP-lacI], MTW1-13xMYC-TRP1, tyr1-1, lys2::pMDE798[P_{DMC1}-GFP-lacI, LYS2], zip1::KanMX6, spo11::KanMX6, CEN1::pJN2[lacO256 LEU2]	**Fig. 2B**
	Y1837: MAT∝, leu2, lys2::pMDE798[P_{DMC1}-GFP-lacI], met13-c, tyr1-2, trp1-Δ63, cyh2-1,	

	spo11::KanMX6, ura3-1, MTW1-13xMYC-his5, his3-Δ1, zip1::pELK12[zip1-NM1], CEN1::pJN2[lacO256 LEU2]	
DEK198	X2065: *MAT***a***, trp1-63, his3-Δ1, leu2, met13-d, ura3-52::pAFS152[URA3* P_{CYC1}*-GFP-lacI], MTW1-13xMYC-TRP1, tyr1-1, lys2::pMDE798[*P_{DMC1}*-GFP-lacI, LYS2], zip1::KanMX6, spo11::KanMX6, CEN1::pJN2[lacO256 LEU2]*<br>Y1838: *MAT∝, leu2, lys2::pMDE798[*P_{DMC1}*-GFP-lacI], met13-c, tyr1-2, trp1-Δ63, cyh2-1, spo11::KanMX6, ura3-1, MTW1-13xMYC-his5, his3-Δ1, zip1::pELK13[zip1-NM2], CEN1::pJN2[lacO256 LEU2]*	**Fig. 2B**
DEK113	X2066: *MAT***a***, trp1-63, his3-Δ1, leu2, met13-d, ura3-52::pAFS152[URA3* P_{CYC1}*-GFP-lacI], MTW1-13xMYC-TRP1, tyr1-1, lys2::pMDE798[*P_{DMC1}*-GFP-lacI, LYS2], zip1::KanMX6, spo11::KanMX6, CEN1::pJN2[lacO256 LEU2]*<br>Y1841: *MAT∝, leu2, lys2::pMDE798[*P_{DMC1}*-GFP-lacI], met13-c, tyr1-2, trp1-Δ63, cyh2-1, spo11::KanMX6, ura3-1, MTW1-13xMYC-his5, his3-Δ1, zip1::pELK15[ZIP1-M1], CEN1::pJN2[lacO256 LEU2]*	**Fig. 2B**
DEK202	X2065: *MAT***a***, trp1-63, his3-Δ1, leu2, met13-d, ura3-52::pAFS152[URA3* P_{CYC1}*-GFP-lacI], MTW1-13xMYC-TRP1, tyr1-1, lys2::pMDE798[*P_{DMC1}*-GFP-lacI, LYS2], zip1::KanMX6, spo11::KanMX6, CEN1::pJN2[lacO256 LEU2]*<br>Y1841: *MAT∝, leu2, lys2::pMDE798[*P_{DMC1}*-GFP-lacI], met13-c, tyr1-2, trp1-Δ63, cyh2-1, spo11::KanMX6, ura3-1, MTW1-13xMYC-his5, his3-Δ1, zip1::pELK15[zip1-M1], CEN1::pJN2[lacO256 LEU2]*	**Fig. 2B**
DEK147	X2066: *MAT***a***, trp1-63, his3-Δ1, leu2, met13-d, ura3-52::pAFS152[URA3* P_{CYC1}*-GFP-lacI], MTW1-13xMYC-TRP1, tyr1-1, lys2::pMDE798[*P_{DMC1}*-GFP-lacI, LYS2], zip1::KanMX6, spo11::KanMX6, CEN1::pJN2[lacO256 LEU2]*<br>Y1848: *MAT∝, leu2, lys2::pMDE798[*P_{DMC1}*-GFP-lacI], met13-c, tyr1-2, trp1-Δ63, cyh2-1, spo11::KanMX6, ura3-1, MTW1-13xMYC-his5, his3-Δ1, zip1::pELK15[zip1-M2], CEN1::pJN2[lacO256 LEU2]*	**Fig. 2B**
DEK203	X2065: *MAT***a***, trp1-63, his3-Δ1, leu2, met13-d, ura3-52::pAFS152[URA3* P_{CYC1}*-GFP-lacI], MTW1-13xMYC-TRP1, tyr1-1, lys2::pMDE798[*P_{DMC1}*-GFP-lacI, LYS2], zip1::KanMX6, spo11::KanMX6, CEN1::pJN2[lacO256 LEU2]*	**Fig. 2B**

	Y1848: *MAT∝, leu2, lys2::pMDE798[*P_{DMC1}*-GFP-lacI], met13-c, tyr1-2, trp1-Δ63, cyh2-1, spo11::KanMX6, ura3-1, MTW1-13xMYC-his5, his3-Δ1, zip1::pELK15[zip1-M2], CEN1::pJN2[lacO256 LEU2]*	
DEK148	X2066: *MAT***a***, trp1-63, his3-Δ1, leu2, met13-d, ura3-52::pAFS152[URA3* P_{CYC1}*-GFP-lacI], MTW1-13xMYC-TRP1, tyr1-1, lys2::pMDE798[*P_{DMC1}*-GFP-lacI, LYS2], zip1::KanMX6, spo11::KanMX6, CEN1::pJN2[lacO256 LEU2]*	**Fig. 2B**
	Y1884: *MAT∝, leu2, lys2::pMDE798[*P_{DMC1}*-GFP-lacI], met13-c, tyr1-2, trp1-Δ63, cyh2-1, spo11::KanMX6, ura3-1, MTW1-13xMYC-his5, his3-Δ1, zip1::pELK16[zip1-MC1], CEN1::pJN2[lacO256 LEU2]*	
DEK204	X2065: *MAT***a***, trp1-63, his3-Δ1, leu2, met13-d, ura3-52::pAFS152[URA3* P_{CYC1}*-GFP-lacI], MTW1-13xMYC-TRP1, tyr1-1, lys2::pMDE798[*P_{DMC1}*-GFP-lacI, LYS2], zip1::KanMX6, spo11::KanMX6, CEN1::pJN2[lacO256 LEU2]*	**Fig. 2B**
	Y1884: *MAT∝, leu2, lys2::pMDE798[*P_{DMC1}*-GFP-lacI], met13-c, tyr1-2, trp1-Δ63, cyh2-1, spo11::KanMX6, ura3-1, MTW1-13xMYC-his5, his3-Δ1, zip1::pELK16[zip1-MC1], CEN1::pJN2[lacO256 LEU2]*	
DEK199	X2065: *MAT***a***, trp1-63, his3-Δ1, leu2, met13-d, ura3-52::pAFS152[URA3* P_{CYC1}*-GFP-lacI], MTW1-13xMYC-TRP1, tyr1-1, lys2::pMDE798[*P_{DMC1}*-GFP-lacI, LYS2], zip1::KanMX6, spo11::KanMX6, CEN1::pJN2[lacO256 LEU2]*	**Fig. 2B**
	Y1842: *MAT∝, leu2, lys2::pMDE798[*P_{DMC1}*-GFP-lacI], met13-c, tyr1-2, trp1-Δ63, cyh2-1, spo11::KanMX6, ura3-1, MTW1-13xMYC-his5, his3-Δ1, zip1::pELK17[zip1-MC2], CEN1::pJN2[lacO256 LEU2]*	
DEK149	X2066: *MAT***a***, trp1-63, his3-Δ1, leu2, met13-d, ura3-52::pAFS152[URA3* P_{CYC1}*-GFP-lacI], MTW1-13xMYC-TRP1, tyr1-1, lys2::pMDE798[*P_{DMC1}*-GFP-lacI, LYS2], zip1::KanMX6, spo11::KanMX6, CEN1::pJN2[lacO256 LEU2]*	**Fig. 2B**
	Y1887: *MAT∝, leu2, lys2::pMDE798[*P_{DMC1}*-GFP-lacI], met13-c, tyr1-2, trp1-Δ63, cyh2-1, spo11::KanMX6, ura3-1, MTW1-13xMYC-his5, his3-Δ1, zip1::pELK18[zip1-C1], CEN1::pJN2[lacO256 LEU2]*	
DEK200	X2065: *MAT***a***, trp1-63, his3-Δ1, leu2, met13-d, ura3-52::pAFS152[URA3* P_{CYC1}*-GFP-lacI], MTW1-13xMYC-TRP1, tyr1-1, lys2::pMDE798[*P_{DMC1}*-GFP-lacI, LYS2], zip1::KanMX6, spo11::KanMX6, CEN1::pJN2[lacO256 LEU2]*	**Fig. 2B**

	Y1887: MAT∝, leu2, lys2::pMDE798[P_{DMC1}-GFP-lacI], met13-c, tyr1-2, trp1-Δ63, cyh2-1, spo11::KanMX6, ura3-1, MTW1-13xMYC-his5, his3-Δ1, zip1::pELK18[zip1-C1], CEN1::pJN2[lacO256 LEU2]	
DEK150	X2066: MAT**a**, trp1-63, his3-Δ1, leu2, met13-d, ura3-52::pAFS152[URA3 P_{CYC1}-GFP-lacI], MTW1-13xMYC-TRP1, tyr1-1, lys2::pMDE798[P_{DMC1}-GFP-lacI, LYS2], zip1::KanMX6, spo11::KanMX6, CEN1::pJN2[lacO256 LEU2]	**Fig. 2B**
	Y1888: MAT∝, leu2, lys2::pMDE798[P_{DMC1}-GFP-lacI], met13-c, tyr1-2, trp1-Δ63, cyh2-1, spo11::KanMX6, ura3-1, MTW1-13xMYC-his5, his3-Δ1, zip1::pELK19[zip1-C2], CEN1::pJN2[lacO256 LEU2]	
DEK205	X2065: MAT**a**, trp1-63, his3-Δ1, leu2, met13-d, ura3-52::pAFS152[URA3 P_{CYC1}-GFP-lacI], MTW1-13xMYC-TRP1, tyr1-1, lys2::pMDE798[P_{DMC1}-GFP-lacI, LYS2], zip1::KanMX6, spo11::KanMX6, CEN1::pJN2[lacO256 LEU2]	**Fig. 2B**
	Y1888: MAT∝, leu2, lys2::pMDE798[P_{DMC1}-GFP-lacI], met13-c, tyr1-2, trp1-Δ63, cyh2-1, spo11::KanMX6, ura3-1, MTW1-13xMYC-his5, his3-Δ1, zip1::pELK19[zip1-C2], CEN1::pJN2[lacO256 LEU2]	
DEK138	X2066: MAT**a**, trp1-63, his3-Δ1, leu2, met13-d, ura3-52::pAFS152[URA3 P_{CYC1}-GFP-lacI], MTW1-13xMYC-TRP1, tyr1-1, lys2::pMDE798[P_{DMC1}-GFP-lacI, LYS2], zip1::KanMX6, spo11::KanMX6, CEN1::pJN2[lacO256 LEU2]	**Fig. 2B**
	Y1849: MAT∝, leu2, lys2::pMDE798[P_{DMC1}-GFP-lacI], met13-c, tyr1-2, trp1-Δ63, cyh2-1, spo11::KanMX6, MTW1-13xMYC-his5, his3-Δ1, ura3-52::pAFS152[URA3 P_{CYC1}-GFP-lacI], zip1::KanMX6, CEN1::pJN2[lacO256 LEU2]	
DEK201	X2065: MAT**a**, trp1-63, his3-Δ1, leu2, met13-d, ura3-52::pAFS152[URA3 P_{CYC1}-GFP-lacI], MTW1-13xMYC-TRP1, tyr1-1, lys2::pMDE798[P_{DMC1}-GFP-lacI, LYS2], zip1::KanMX6, spo11::KanMX6, CEN1::pJN2[lacO256 LEU2]	**Fig. 2B**
	Y1849: MAT∝, leu2, lys2::pMDE798[P_{DMC1}-GFP-lacI], met13-c, tyr1-2, trp1-Δ63, cyh2-1, spo11::KanMX6, MTW1-13xMYC-his5, his3-Δ1, ura3-52::pAFS152[URA3 P_{CYC1}-GFP-lacI], zip1::KanMX6, CEN1::pJN2[lacO256 LEU2]	
DEK263	X2268: MAT**a**, ura3-13, trp1-63, leu2, leu2::OPL46[P_{URA3}-tetR-tdTomato leu2::HIS3], met13-d, tyr1-1, can1-R, lys2::pMDE798[P_{DMC1}-GFP-lacI, LYS2], zip1::KanMX6, ura3::pKB80	**Fig. 3A, B**

	[P_{GPD1}-GAL4(848)-ER-URA3::hphNT1], natNT2-P_{GAL1}-NDT80, OPL210[CEN3-5.1KB, lacO256, LEU2 TRP1, ARS1], OPL214[CEN3-5.1KB, tetO256, URA3 TRP1, ARS1]	
	Y2056: MAT∝, leu2, lys2::pMDE798[P_{DMC1}-GFP-lacI], met13-c, tyr1-2, trp1-Δ63, cyh2-1, ura3-1, his3-Δ1, zip1::pELK10[ZIP1], natNT2-P_{GAL1}-NDT80	
DEK264	X2268: MAT**a**, ura3-13, trp1-63, leu2, leu2::OPL46[P_{URA3}-tetR -tdTomato leu2::HIS3], met13-d, tyr1-1, can1-R, lys2::pMDE798[P_{DMC1}-GFP-lacI, LYS2], zip1::KanMX6, ura3::pKB80 [P_{GPD1}-GAL4(848)-ER-URA3::hphNT1], natNT2-P_{GAL1}-NDT80, OPL210[CEN3-5.1KB, lacO256, LEU2 TRP1, ARS1], OPL214[CEN3-5.1KB, tetO256, URA3 TRP1, ARS1]	**Fig. 3A, B**
	Y2038: MAT∝, leu2, lys2::pMDE798[P_{DMC1}-GFP-lacI], met13-c, tyr1-2, trp1-Δ63, cyh2-1, ura3-1, his3-Δ1, zip1::pELK11[zip1-N1], natNT2-P_{GAL1}-NDT80	
DEK285	X2295: MAT∝, ura3-13, trp1-63, leu2, leu2::OPL46[P_{URA3}-tetR -tdTomato leu2::HIS3], met13-d, tyr1-1, can1-R, lys2::pMDE798[P_{DMC1}-GFP-lacI, LYS2], zip1::KanMX6, ura3::pKB80 [P_{GPD1}-GAL4(848)-ER-URA3::hphNT1], natNT2-P_{GAL1}-NDT80, OPL210[CEN3-5.1KB, lacO256, LEU2 TRP1, ARS1], OPL214[CEN3-5.1KB, tetO256, URA3 TRP1, ARS1]	**Fig. 3B**
	Y2055: MAT**a**, leu2, lys2::pMDE798[P_{DMC1}-GFP-lacI], met13-c, tyr1-2, trp1-Δ63, cyh2-1, ura3-1, his3-Δ1, zip1::pELK10[ZIP1], natNT2-P_{GAL1}-NDT80	
DEK286	X2295: MAT∝, ura3-13, trp1-63, leu2, leu2::OPL46[P_{URA3}-tetR -tdTomato leu2::HIS3], met13-d, tyr1-1, can1-R, lys2::pMDE798[P_{DMC1}-GFP-lacI, LYS2], zip1::KanMX6, ura3::pKB80 [P_{GPD1}-GAL4(848)-ER-URA3::hphNT1], natNT2-P_{GAL1}-NDT80, OPL210[CEN3-5.1KB, lacO256, LEU2 TRP1, ARS1], OPL214[CEN3-5.1KB, tetO256, URA3 TRP1, ARS1]	**Fig. 3B**
	Y2037: MAT**a**, leu2, lys2::pMDE798[P_{DMC1}-GFP-lacI], met13-c, tyr1-2, trp1-Δ63, cyh2-1, ura3-1, his3-Δ1, zip1::pELK11[zip1-N1], natNT2-P_{GAL1}-NDT80	
DEK265	X2268: MAT**a**, ura3-13, trp1-63, leu2, leu2::OPL46[P_{URA3}-tetR -tdTomato leu2::HIS3], met13-d, tyr1-1, can1-R, lys2::pMDE798[P_{DMC1}-GFP-lacI, LYS2], zip1::KanMX6, ura3::pKB80 [P_{GPD1}-GAL4(848)-ER-URA3::hphNT1], natNT2-P_{GAL1}-NDT80, OPL210[CEN3-5.1KB, lacO256,	**Fig. 3B**

	LEU2 TRP1, ARS1], OPL214[CEN3-5.1KB, tetO256, URA3 TRP1, ARS1]<br>Y2040: MAT∝, leu2, lys2::pMDE798[P_{DMC1}-GFP-lacI], met13-c, tyr1-2, trp1-Δ63, cyh2-1, ura3-1, his3-Δ1, zip1::pELK12[zip1-NM1], natNT2-P_{GAL1}-NDT80	
DEK287	X2295: MAT∝, ura3-13, trp1-63, leu2, leu2::OPL46[PURA3-tetR-tdTomato leu2::HIS3], met13-d, tyr1-1, can1-R, lys2::pMDE798[P_{DMC1}-GFP-lacI, LYS2], zip1::KanMX6, ura3::pKB80 [P_{GPD1}-GAL4(848)-ER-URA3::hphNT1], natNT2-P_{GAL1}-NDT80, OPL210[CEN3-5.1KB, lacO256, LEU2 TRP1, ARS1], OPL214[CEN3-5.1KB, tetO256, URA3 TRP1, ARS1]<br>Y2039: MAT**a**, leu2, lys2::pMDE798[P_{DMC1}-GFP-lacI], met13-c, tyr1-2, trp1-Δ63, cyh2-1, ura3-1, his3-Δ1, zip1::pELK12[zip1-NM1], natNT2-P_{GAL1}-NDT80	**Fig. 3B**
DEK276	X2268: MAT**a**, ura3-13, trp1-63, leu2, leu2::OPL46[P_{URA3}-tetR -tdTomato leu2::HIS3], met13-d, tyr1-1, can1-R, lys2::pMDE798[P_{DMC1}-GFP-lacI, LYS2], zip1::KanMX6, ura3::pKB80 [P_{GPD1}-GAL4(848)-ER-URA3::hphNT1], natNT2-P_{GAL1}-NDT80, OPL210[CEN3-5.1KB, lacO256, LEU2 TRP1, ARS1], OPL214[CEN3-5.1KB, tetO256, URA3 TRP1, ARS1]<br>Y2044: MAT∝, leu2, lys2::pMDE798[P_{DMC1}-GFP-lacI, LYS2], met13-c, tyr1-2, trp1-Δ63, cyh2-1, ura3-1, his3-Δ1, zip1::pELK15[zip1-M1], natNT2-P_{GAL1}-NDT80	**Fig. 3B**
DEK289	X2295: MAT∝, ura3-13, trp1-63, leu2, leu2::OPL46[PURA3-tetR-tdTomato leu2::HIS3], met13-d, tyr1-1, can1-R, lys2::pMDE798[P_{DMC1}-GFP-lacI, LYS2], zip1::KanMX6, ura3::pKB80 [P_{GPD1}-GAL4(848)-ER-URA3::hphNT1], natNT2-P_{GAL1}-NDT80, OPL210[CEN3-5.1KB, lacO256, LEU2 TRP1, ARS1], OPL214[CEN3-5.1KB, tetO256, URA3 TRP1, ARS1]<br>Y2043: MAT**a**, leu2, lys2::pMDE798[P_{DMC1}-GFP-lacI], met13-c, tyr1-2, trp1-Δ63, cyh2-1, ura3-1, his3-Δ1, zip1::pELK15[zip1-M1], natNT2-P_{GAL1}-NDT80	**Fig. 3B**
DEK277	X2268: MAT**a**, ura3-13, trp1-63, leu2::OPL46[P_{URA3}-tetR -tdTomato leu2::HIS3], met13-d, tyr1-1, can1-R, lys2::pMDE798[P_{DMC1}-GFP-lacI, LYS2], zip1::KanMX6, ura3::pKB80 [P_{GPD1}-GAL4(848)-ER-URA3::hphNT1], natNT2-P_{GAL1}-NDT80, OPL210[CEN3-5.1KB, lacO256, LEU2 TRP1, ARS1], OPL214[CEN3-5.1KB, tetO256, URA3 TRP1, ARS1]	**Fig. 3B**

	Y2046: MAT∝, leu2, lys2::pMDE798[P_{DMC1}-GFP-lacI], met13-c, tyr1-2, trp1-Δ63, cyh2-1, ura3-1, his3-Δ1, zip1::pELK15[zip1-M2], natNT2-P_{GAL1}-NDT80	
DEK278	X2268: MAT**a**, ura3-13, trp1-63, leu2::OPL46[P_{URA3}-tetR -tdTomato leu2::HIS3], met13-d, tyr1-1, can1-R, lys2::pMDE798[P_{DMC1}-GFP-lacI, LYS2], zip1::KanMX6, ura3::pKB80 [P_{GPD1}-GAL4(848)-ER-URA3::hphNT1], natNT2-P_{GAL1}-NDT80, OPL210[CEN3-5.1KB, lacO256, LEU2 TRP1, ARS1], OPL214[CEN3-5.1KB, tetO256, URA3 TRP1, ARS1]	**Fig. 3B**
	Y2048: MAT∝, leu2, lys2::pMDE798[P_{DMC1}-GFP-lacI], met13-c, tyr1-2, trp1-Δ63, cyh2-1, ura3-1, his3-Δ1, zip1::pELK16[zip1-MC1], natNT2-P_{GAL1}-NDT80	
DEK291	X2295: MAT∝, ura3-13, trp1-63, leu2::OPL46[PURA3-tetR-tdTomato leu2::HIS3], met13-d, tyr1-1, can1-R, lys2::pMDE798[P_{DMC1}-GFP-lacI, LYS2], zip1::KanMX6, ura3::pKB80 [P_{GPD1}-GAL4(848)-ER-URA3::hphNT1], natNT2-P_{GAL1}-NDT80, OPL210[CEN3-5.1KB, lacO256, LEU2 TRP1, ARS1], OPL214[CEN3-5.1KB, tetO256, URA3 TRP1, ARS1]	**Fig. 3B**
	Y2047: MAT**a**, leu2, lys2::pMDE798[P_{DMC1}-GFP-lacI], met13-c, tyr1-2, trp1-Δ63, cyh2-1, ura3-1, his3-Δ1, zip1::pELK16[zip1-MC1], natNT2-P_{GAL1}-NDT80	
DEK279	X2268: MAT**a**, ura3-13, trp1-63, , leu2::OPL46[P_{URA3}-tetR -tdTomato leu2::HIS3], met13-d, tyr1-1, can1-R, lys2::pMDE798[P_{DMC1}-GFP-lacI, LYS2], zip1::KanMX6, ura3::pKB80 [P_{GPD1}-GAL4(848)-ER-URA3::hphNT1], natNT2-P_{GAL1}-NDT80, OPL210[CEN3-5.1KB, lacO256, LEU2 TRP1, ARS1], OPL214[CEN3-5.1KB, tetO256, URA3 TRP1, ARS1]	**Fig. 3B**
	Y2050: MAT∝, leu2, lys2::pMDE798[P_{DMC1}-GFP-lacI], met13-c, tyr1-2, trp1-Δ63, cyh2-1, ura3-1, his3-Δ1, zip1::pELK17[zip1-MC2], natNT2-P_{GAL1}-NDT80	
DEK264	X2268: MAT**a**, ura3-13, trp1-63, leu2::OPL46 P_{URA3}-tetR -tdTomato leu2::HIS3], met13-d, tyr1-1, can1-R, lys2::pMDE798[P_{DMC1}-GFP-lacI, LYS2], zip1::KanMX6, ura3::pKB80 [P_{GPD1}-GAL4(848)-ER-URA3::hphNT1], natNT2-P_{GAL1}-NDT80, OPL210[CEN3-5.1KB, lacO256, LEU2 TRP1, ARS1], OPL214[CEN3-5.1KB, tetO256, URA3 TRP1, ARS1]	**Fig. 4A, B, C**
	Y2038: MAT∝, leu2, lys2::pMDE798[P_{DMC1}-GFP-lacI], met13-c, tyr1-2, trp1-Δ63, cyh2-1, ura3-1,	

	his3-Δ1, zip1::pELK11[zip1-N1], natNT2-P_{GAL1}*-NDT80*	
DEK306	X2187: *MAT***a***, ura3-13, trp1-63, leu2::OPL46* P_{URA3}*-tetR -tdTomato leu2::HIS3], met13-d, tyr1-1, can1-R, lys2::pMDE798[*P_{DMC1}*-GFP-lacI, LYS2],* OPL210[*CEN3-5.1KB, lacO256, LEU2 TRP1, ARS1*], OPL214[*CEN3-5.1KB, tetO256, URA3 TRP1, ARS1*]	**Fig. 4B, C**
	Y2009: *MAT∝,trp1-Δ63, his3-Δ1, leu2, lys2::pMDE798[*P_{DMC1}*-GFP-lacI], tyr1-2, met13-c, cyh2-1*	
DHC413B	X3055: *MAT∝, ura3-13, trp1-63, met13-d, tyr1-1, can1-R, zip4::natNT2, MTW1-13xMYC-TRP1, leu2, zip1::KanMX66, ndt80::LEU2*	**Fig. 5A, C**
	Y2707: *MAT***a***, leu2, lys2-2, tyr1-2, met13-c, trp1-Δ63, cyh2-1, his3-Δ1, MTW1-13xMYC-HIS3, ndt80::LEU2, zip4::natNT2*	
DHC412B	X3055: *MAT∝, ura3-13, trp1-63, met13-d, tyr1-1, can1-R, zip4::natNT2, MTW1-13xMYC-TRP1, leu2, zip1::KanMX66, ndt80::LEU2*	**Fig. 5B, C**
	Y2697: *MAT***a***, leu2, met13-c, tyr1-2, trp1-Δ63, cyh2-1, ura3-1, his3-Δ1, zip1::pELK11[zip1-N1], zip4::KanMX66, MTW1-13xMYC-HIS3MX, ndt80::LEU2*	
DHC412A	X3054: *MAT***a***, ura3-13, trp1-63, met13-d, tyr1-1, can1-R, zip4::natNT2, MTW1-13xMYC-TRP1, leu2, zip1::KanMX66, ndt80::LEU2*	**Fig. 5D**
	Y2699: *MAT∝, leu2, met13-c, tyr1-2, trp1-Δ63, cyh2-1, ura3-1, his3-Δ1, zip1::pELK11[zip1-N1], zip4:: :KanMX66, MTW1-13xMYC-HIS3MX6, ndt80::LEU2*	
DHC413A	X3054: *MAT***a***, ura3-13, trp1-63, met13-d, tyr1-1, can1-R, zip4::natNT2, MTW1-13xMYC-TRP1, leu2, zip1:: :KanMX66, ndt80::LEU2*	**Fig. 5D**
	Y2708: *MAT∝, leu2, lys2-2, tyr1-2, met13-c, trp1-Δ63, cyh2-1, his3-Δ1, MTW1-13xMYC-HIS3, ndt80::LEU2, zip4::NatNT2*	
DHC414A	X3054: *MAT***a***, ura3-13, trp1-63, met13-d, tyr1-1, can1-R, zip4::natNT2, MTW1-13xMYC-TRP1, leu2, zip1:: :KanMX66, ndt80::LEU2*	**Fig. 5D**
	Y2701: *MAT∝, leu2, lys2-2, tyr1-2, met13-c, trp1-Δ63, cyh2-1, his3-Δ1, MTW1-13xMYC-HIS3, zip4::natNT2, zip1: :KanMX66, ndt80::LEU2*	
DHC370	Y2507: *MAT***a***, leu2, met13-c, tyr1-2, trp1-Δ63, cyh2-1, ura3-1, MTW1-13xMYC-HIS3MX6, his3-Δ1*	**Fig. S1A**
	Y2508: *MAT∝, leu2, met13-c, tyr1-2, trp1-Δ63, cyh2-1, ura3-1, MTW1-13xMYC-HIS3MX6, his3-Δ1*	

DHC372	X2945: *MAT***a**, *ura3-13, trp1-63, met13-d, tyr1-1, can1-R, MTW1-13xMYC-TRP1, leu2, zip1::KanMX66*	**Fig. S1B**
	X2946: *MAT∝, ura3-13, trp1-63, met13-d, tyr1-1, can1-R, MTW1-13xMYC-TRP1, leu2, zip1::KanMX66*	
DHC371	Y2627: *MAT∝, leu2, met13-c, tyr1-2, trp1-Δ63, cyh2-1, ura3-1, his3-Δ1, zip1::pELK11[zip1-N1], MTW1-13xMYC-HIS3MX6*	**Fig. S1C**
	Y2628: *MAT***a**, *leu2, met13-c, tyr1-2, trp1-Δ63, cyh2-1, ura3-1, his3-Δ1, zip1::pELK11[zip1-N1], MTW1-13xMYC-HIS3MX6*	
DHC373	DHC363.7A: *MAT***a**, *leu2, met13-c, tyr1-2, trp1-Δ63, cyh2-1, ura3-1, his3-Δ1, zip1::pELK11[zip1-N1]*	**Fig. S2D**
	DHC364.10D: *MAT∝, leu2, met13-c, tyr1-2, trp1-Δ63, cyh2-1, ura3-1, his3-Δ1, zip1::pELK11[zip1-N1]*	
DJE77	X2946: *MAT∝, ura3-13, trp1-63, met13-d, tyr1-1, can1-R, MTW1-13xMYC-TRP1, leu2-?, zip1::KANMX*	**Fig. S2A-C**
	Y1941: *MAT***a**, *leu2, lys2::pMDE798[PDMC1-GFP-lacI], met13-c, tyr1-2, trp1-Δ63, cyh2-1, ura3-1, his3-Δ1, zip1::pELK10[ZIP1]*	
DJE78	X2946: *MAT∝, ura3-13, trp1-63, met13-d, tyr1-1, can1-R, MTW1-13xMYC-TRP1, leu2-?, zip1::KANMX*	**Fig. S2A-C**
	Y2001: *MAT***a**, *leu2, lys2::pMDE798[PDMC1-GFP-lacI], met13-c, tyr1-2, trp1-Δ63, cyh2-1, ura3-1, his3-Δ1, zip1::pELK11[zip1-N1]*	
DJE72	X2946: *MAT∝, ura3-13, trp1-63, met13-d, tyr1-1, can1-R, MTW1-13xMYC-TRP1, leu2-?, zip1::KANMX*	**Fig. S2C**
	Y2027: *MAT***a**, *trp1-Δ63, his3-Δ1, leu2, lys2::pMDE798[PDMC1-GFP-lacI], tyr1-2, met13-c, cyh2-1, zip1::KANMX*	
DHC374	Y169: *MAT∝, leu2, lys2-2, met13-c, tyr1-2, ura3-1, trp1-Δ63, cyh2-1, his3-Δ1*	**Fig. S2D**
	Y170: *MAT***a**, *leu2, lys2-2, met13-c, tyr1-2, ura3-1, trp1-Δ63, cyh2-1, his3-Δ1*	
DJE84	Y1941: *MATa, leu2, lys2::pMDE798[*P_{DMC1}*-GFP-lacI], met13-c, tyr1-2, trp1-Δ63, cyh2-1, ura3-1, his3-Δ1, zip1::pELK10[ZIP1]*	**Fig. S3**
	Y1942: *MAT∝, leu2, lys2::pMDE798[*P_{DMC1}*-GFP-lacI], met13-c, tyr1-2, trp1-Δ63, cyh2-1, ura3-1, his3-Δ1, zip1::pELK10[ZIP1]*	
DJE85	Y2001: *MATa, leu2, lys2::pMDE798[*P_{DMC1}*-GFP-lacI], met13-c, tyr1-2, trp1-Δ63, cyh2-1, ura3-1, his3-Δ1, zip1::pELK11[zip1-N1]*	**Fig. S3**

	Y2627: *MAT∝, leu2, met13-c, tyr1-2, trp1-Δ63, cyh2-1, ura3-1, his3-Δ1, zip1::pELK11[zip1-N1], MTW1-13xMYC-HIS3MX6*	
