# Supplementary material for: A ZIP1 separation-of-function allele reveals that centromere pairing drives meiotic segregation of achiasmate chromosomes in budding yeast
Source: PLoS Genet. 2018 Aug 9;14(8):e1007513. doi: 10.1371/journal.pgen.1007513 (PMC6103513; doi:10.1371/journal.pgen.1007513)
Supplement: S2 Table — (PDF) [file pgen.1007513.s005.pdf]

Table S2. Statistics for centromere coupling experiments

genotype	number of spreads scored	average number of Mtw1 foci	standard deviation	P value vs *ZIP1*	P value vs *zip1Δ*
ZIP1	207	16.45	3.16		****
zip1-N1	165	15.90	2.51	ns	****
zip1-NM1	100	18.69	3.74	****	ns
zip1-NM2	200	18.76	3.98	****	ns
zip1-M1	153	17.18	3.60	ns	****
zip1-N2	152	17.56	3.49	*	**
zip1-MC1	155	16.97	3.65	ns	****
zip1-MC2	104	16.09	3.12	ns	****
zip1-C1	303	19.41	3.69	****	ns
zip1-C2	254	16.53	3.37	ns	****
zip1Δ	257	19.19	3.77	****	

Significance was evaluated with the Kruskal-Wallis test with Dunn's correction for multiple comparisons, performed using Prism 6.0.

ns, P>0.05, * P ≤ 0.05, ** P ≤ 0.01, *** P ≤ 0.001, **** P ≤ 0.0001
